# Circular RNAs in Neurons: From Transport to Function

**DOI:** 10.3390/ncrna12040027

**Published:** 2026-07-29

**Authors:** Nicolò Salvi, Mariangela Morlando

**Affiliations:** 1 Department of Biology and Biotechnologies “Charles Darwin”, Sapienza University of Rome, 00185 Rome, Italy; n.salvi@uniroma1.it; 2 Center for Life Nano- and Neuro-Science, Istituto Italiano di Tecnologia, 00161 Rome, Italy

**Keywords:** circular RNA, localization, transport, neurons, synapses, axons, neuronal disorders

## Abstract

Circular RNAs (circRNAs) have recently emerged as a class of abundant and remarkably stable non-coding RNAs preferentially enriched in the nervous system. In neurons, the fine-tuned spatial regulation of gene expression is critical for proper synaptic function; accordingly, several studies have demonstrated that circRNAs exhibit highly compartmentalized localization, specifically within dendrites, axons, and synapses. These spatial localization properties imply the presence of active transport mechanisms, which control the intracellular trafficking of circRNAs. This review highlights the current understanding of circRNA transport in neurons, focusing on the molecular machinery driving synaptic enrichment. We explore the potential role of ribonucleoprotein-based transport as a primary mechanism driving circRNA localization and examine how such spatial distribution influences synaptic plasticity and post-transcriptional gene regulation. Finally, we discuss the clinical implications of these processes, exploring the link between dysregulated RNA transport and the development of neuronal abnormalities.

## 1. Introduction

Circular RNAs (circRNAs) belong to a large family of transcripts generated through back-splicing, a process that produces covalently closed loop structures devoid of 5′ caps and 3′ poly(A) tails [1]. This unique architecture renders them exceptionally resistant to exonucleolytic degradation, granting them significantly higher stability than their linear counterparts. Consequently, circRNAs are remarkably abundant in long-lived, post-mitotic cell types, such as neurons [2]. Initially perceived as by-products of aberrant alternative splicing, circRNAs are now recognized as evolutionarily conserved, highly abundant RNA species endowed with critical regulatory capabilities.

A characteristic feature of the neuronal transcriptome is its vast repertoire of circRNAs. Indeed, recent studies demonstrate that mammalian brain tissues harbor a greater diversity and abundance of circRNAs than any other organ [3]. Crucially, these molecules accumulate progressively during neural development and aging, persisting long after cell division ceases [4,5]. Notably, circRNAs are not homogeneously distributed within neuronal cells; rather, they display strict subcellular compartmentalization, concentrating within dendrites, axons, and synaptic terminals. Transcriptomic and biochemical approaches show the enrichment of circRNAs in neuropile and synaptosome fractions, suggesting a tight correlation between localized circRNAs and host genes encoding core synaptic components [6,7,8]. Because the structural and functional integrity of synapses is critical for both synaptic plasticity and neurodevelopment [9], synaptic dysfunction has emerged as a common hallmark of neurodevelopmental disorders, such as autism and epilepsy [10], as well as neurodegenerative diseases, including Alzheimer’s (AD) and Parkinson’s disease (PD) [11]. Unsurprisingly, the dysregulation of circRNA expression has been increasingly described across these pathological contexts [12,13].

Given the extreme polarization of neurons and the significant physical distances between the soma and distal projections, precise RNA distribution is crucial for rapid and site-specific gene expression. While several well-studied pathways governing messenger RNA (mRNA) distribution in neurons (including transport, localization, local translation, and degradation) have been described [14,15], the mechanisms dictating the intracellular movement of circRNAs remain poorly understood. Nonetheless, the marked accumulation of circRNAs in distal projections raises pivotal questions regarding their active transport, localization, and retention in specific cellular sites.

Emerging evidence suggests that circRNAs associate with specific RNA-binding proteins (RBPs) to form ribonucleoprotein (RNP) complexes capable of using the canonical cytoskeleton-mediated transport machinery [16]. For instance, circRNAs are hypothesized to utilize kinesin and dynein motor complexes for long-distance trafficking along microtubules and actin-dependent local movement within dendritic spines and synaptic domains. Additionally, circRNAs appear to be actively incorporated into dynamic RNA granules and biomolecular condensates.

Elucidating the precise mechanisms regulating circRNA transport and localization is essential to decoding their functional roles in neuronal physiology. This review highlights recent advances in circRNA trafficking, explores their targeted delivery to synaptic sites and discusses their emerging implications in neurological disorders.

## 2. CircRNAs in the Brain: Abundance and Spatial Distribution

### 2.1. Molecular Drivers of Neuronal Accumulation

CircRNAs are highly enriched in the nervous system compared to other tissues, displaying distinct profiles across discrete brain regions [7]. Several physiological features may contribute to this neural enrichment. The covalently closed structure confers high stability to circRNAs, allowing them to accumulate in post-mitotic neurons that undergo minimal turnover compared to proliferative cell types [17,18]. These cell types display profound transcriptional activity and highly complex alternative splicing programs, which favor back-splicing events. Moreover, neural genes are characterized by exceptionally long introns, a feature known to facilitate flanking intronic base-pairing that promotes circularization [19].

### 2.2. Temporal Dynamics and Synaptic Localization

The temporal expression of circRNAs is tightly coupled to neuronal differentiation, synaptogenesis, and aging [4,5]. During development, the neuronal circRNA profile undergoes dramatic remodeling displaying a significant increase during early postnatal development, coinciding with the peak of synaptogenesis [6]. Many brain-enriched circRNAs derive from host genes involved in synaptic scaffolding and plasticity, further highlighting a relationship between circRNA expression and function and neuronal physiology.

Beyond temporal regulation, spatial compartmentalization is a hallmark of the neuronal circRNA landscape. Indeed, many circRNAs have been actively sorted into dendrites and synaptoneurosomes. Crucially, this synaptic localization is modulated by neuronal activity. Stimuli that trigger synaptic plasticity prompt rapid, activity-dependent alterations in both the abundance and local positioning of specific circRNAs [6,20].

Prominent examples of a functionally validated neural circRNA are *CDR1as* (also designated ciRS-7) and *Circ-Dlc1(2)*. Genetic ablation of either of these circRNA in mouse models causes alterations in synaptic transmission, defective vesicular release, and distinct behavioral phenotypes, firmly highlighting their functional relevance within mature neuronal circuits [20,21].

The enrichment of circRNAs within distal compartments raises important questions regarding how these molecules are transported to specific sites. Passive diffusion is physically insufficient to meet the rapid spatial demands of the synapse. Instead, this polarized distribution suggests the involvement of an active, highly organized transport network. The association of many circRNAs with neural RBPs strongly implies that these circular transcripts are packaged into distinct RNP complexes that may allow them to engage active intracellular transport pathways [22].

## 3. Mechanisms of Neuronal circRNA Transport

While the mechanisms controlling mRNA localization and local translation have been investigated for decades, the rules governing circRNA trafficking within neurons are only beginning to be elucidated. Current evidence supports a model wherein circRNAs are incorporated into neuronal RNP granules via coordinated interactions with RBPs.

**Figure 1:**
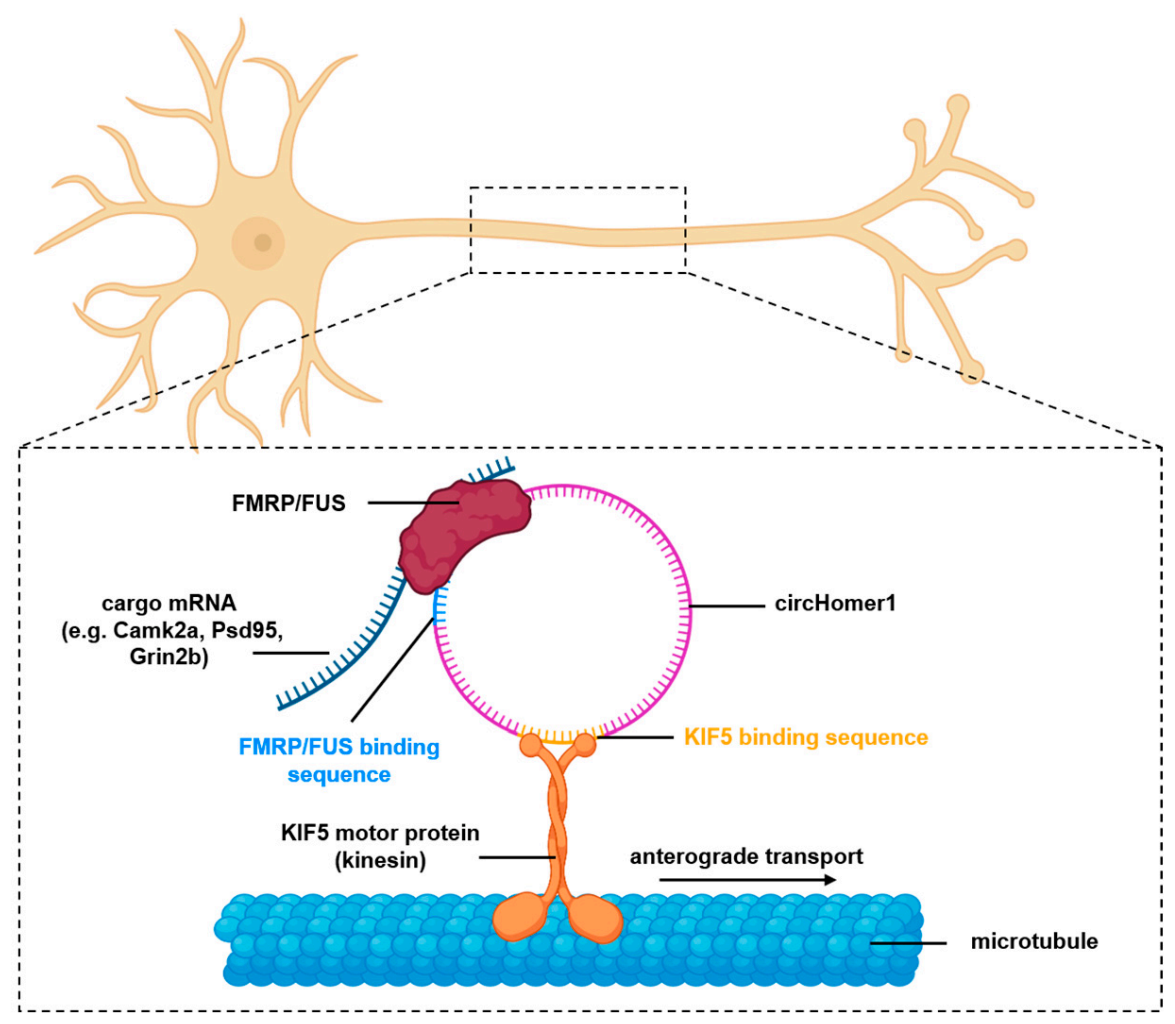
Schematic representation of *circHomer1* trafficking within neurons. *CircHomer1* associates with RBPs such as FUS and FMRP, forming RNP complexes that are transported along dendritic microtubules by kinesin motor proteins.

A prime example of this mechanism involves the highly abundant neuronal circRNA *circHomer1*, a circRNA derived from the synaptic scaffolding gene *Homer1*. *CircHomer1* is highly expressed in dendrites and synaptic compartments where it modulates synaptic plasticity genes [23]; its expression and positioning at synaptic sites are regulated by neuronal activity and by physical interaction with neuronal RNP granules. Recent work by Cai et al. [16] demonstrated that *circHomer1* physically associates with the RNA-binding proteins FMRP (Fragile X Messenger Ribonucleoprotein) and FUS (Fused in -sarcoma), two components of neuronal RNP granules. Through these interactions, *circHomer1* promotes the formation of RNP granules and the association with the kinesin-1 motor protein KIF5. These associations are mediated through predicted regions within the circRNA sequence, which enable *circHomer1* to simultaneously bind to RNP granule proteins and motor machinery. These interactions enable dendritic trafficking of synaptic mRNAs, including *Camk2a*, *Psd95* and *Grin2b*, which encode proteins essential for postsynaptic organization, receptor signaling and synaptic plasticity (Figure 1). Depletion of *circHomer1* disrupts the interaction between RNP granules and KIF5, reducing the dendritic localization of these transcripts without affecting their expression levels. Consequently, the local translation of synaptic proteins is impaired, leading to defects in synaptic transmission. This mechanism provides evidence that circRNAs can actively regulate neuronal transport rather than simply localizing at synapses.

Beyond *circHomer1*, other neuronal circRNAs utilize the RBP-motor interface to achieve precise dendritic localization. For instance, HuD (ELAVL4), a RBP involved in neuronal differentiation, regeneration, synaptic plasticity and learning and memory, has been described to binds to several circRNAs expressed in mouse striatum and among them *circUfp2,* which is enriched in synaptosomes [24]. Given that HuD physically interacts with the Survival of Motor Neuron (SMN) protein to form axonal RNP transport complexes that govern the spatial distribution of synaptic linear mRNAs [25], it is plausible that a conserved macromolecular mechanism is utilized to traffic synapse-enriched circRNAs.

More broadly, several RBPs have been associated with circRNA biology, including FMRP, ELAV/Hu proteins, FUS and hnRNP family members [16,26,27]. These proteins are known regulators of linear RNA transport and likely serve dual roles in sorting circRNAs into localized transport granules.

Despite these insights, fundamental aspects of circRNA transport remain elusive. It remains to be determined whether circRNAs contain unique localization signals analogous to the “zip codes” identified in mRNAs [28] or if their spatial sorting is determined purely by structural conformations, which influence RNP assembly and trafficking. Nevertheless, current findings strongly support a model where circRNAs are integrated into canonical RNA transport pathways through RBP-mediated interactions and cytoskeletal trafficking systems.

## 4. Functional Roles of circRNAs in Synaptic Regulation

The enrichment of circRNAs within synaptic compartments strongly points to their active contribution to local regulatory networks that sustain synaptic function. Synapses require rapid and decentralized gene expression to support neurotransmission, structural remodeling, and long-term plasticity. In polarized neurons, these processes rely on localized RNA metabolism, including transport, translation, and targeted degradation. Yet, while high-throughput profiling has revealed an abundance of synapse-enriched circRNAs, the precise molecular mechanisms have been detailed for only a small subset.

**Figure 2:**
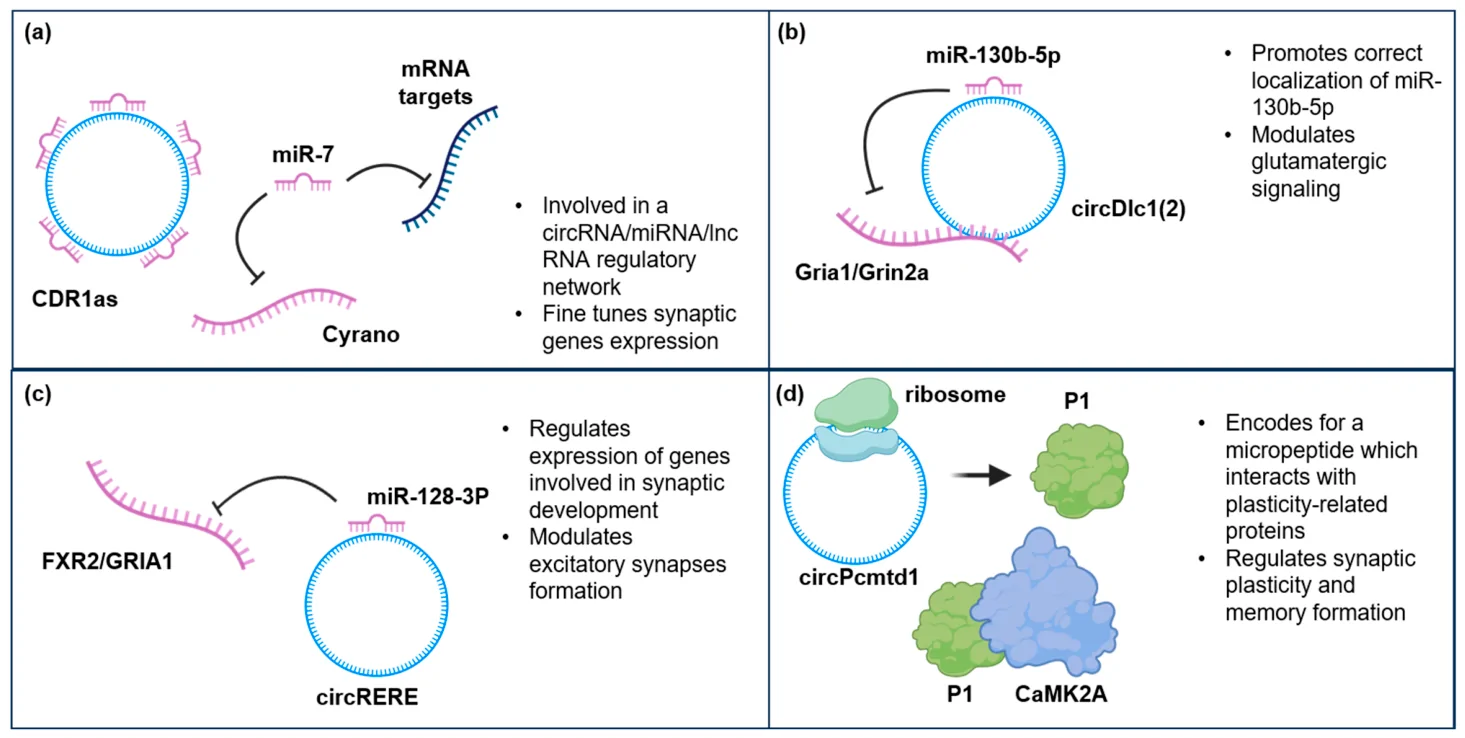
Schematic overview of representative circRNAs implicated in neuronal and synaptic functions: (**a**) *CDR1as* modulates the *miR-7* regulatory network, along with the lncRNA *Cyrano*, tuning the expression of synaptic genes; (**b**) *CircDlc1(2)* localizes *miR-130b-5p* at the synapse and, synergistically, they downregulate genes involved in glutamatergic signaling; (**c**) *CircRERE* stabilizes *miR-128-3p,* leading to downregulation of genes related to the formation of excitatory synapses; (**d**) *CircPcmtd1* contains an IRES that encodes for a locally translated micro-peptide which is able to interact with proteins related to synaptic plasticity, modulating fear extinction memory.

One of the most described functions of circRNAs is their ability to interact with microRNAs (miRNAs). Several circRNAs contain multiple miRNA-binding sites and can act as molecular sponges to modulate miRNA availability and downstream target activity. The classic paradigm of this mechanism is *CDR1as*, which contains numerous binding sites for *miR-7* (130 in mice and over 70 in humans [29]) and regulates its availability, thus modulating pathways crucial for homeostatic neuronal activity. This axis is further integrated into a broader non-coding RNA network via the long non-coding RNA (lncRNA) *Cyrano,* which promotes the targeted degradation of *miR-7* to indirectly regulate *CDR1as* signaling (Figure 2a). Disruption of this network leads to alterations in neuronal excitability and synaptic plasticity [20]. A more recent study by Cerda-Jara et al. (2024) [30] revealed that *Cdr1as* co-localizes within synaptic compartments, demonstrating that the *Cdr1as–miR-7* complex is homogeneously distributed along neuronal projections under physiological conditions. Additionally, selective knockdown of *Cdr1as* specifically within cortical neuronal processes reduces local *miR-7a* and *miR-7b* levels and subsequently impairs memory extinction [31]. While these findings firmly confirm that this circular transcript exerts localized regulatory control over synaptic functions [20,30], they also reshape the historical classification of *CDR1as*, shifting its identity from a canonical miRNA sponge to an active stabilizer and transporter of *miR-7* molecules.

Beyond direct sponging to activate downstream targets, emerging evidence highlights a more multifaceted role for circRNAs at the periphery. This regulatory versatility is represented by *circDlc1(2)*, a circRNA characterized in the murine striatum, able to coordinate a synergistic repressive complex. It contains binding sites for *miR-130b-5p* alongside binding motifs for *Gria1* and *Grin2a*, two essential mRNAs involved in glutamatergic signaling. Notably, *Gria1* and *Grin2a* are also targeted by *miR-130b-5p*. Rather than acting as a simple competitive inhibitor, *circDlc1(2)* works cooperatively with *miR-130b-5p* to repress these glutamatergic targets (Figure 2b), thereby controlling neuronal glutamate uptake. Crucially, Silenzi et al. demonstrated that *circDlc1(2)* controls the spatial localization of *miR-130b-5p* at the neuronal periphery, where the targets reside, ensuring strict compartmentalization of gene expression [21].

Shifting focus from mature neurotransmission to synaptogenesis, another example of a circRNA–miRNA axis is represented by *circRERE*. This circRNA directly interacts with *miR-128-3p*, protecting it from the canonical decay pathway. This stabilization ensures the appropriate translational repression of specific downstream synaptic target mRNAs. Among them is *FXR2*, which promotes the activity-dependent local translation of Postsynaptic Density Protein 95, PSD95, a fundamental scaffolding component of excitatory postsynaptic architectures, and *Gria1*, encoding the AMPA-type glutamate receptor subunit GluA1, a principal determinant of dendritic spine morphology and excitatory transmission (Figure 2c). Collectively, the homeostatic function of *circRERE* is to act as a molecular constraint on aberrant synaptogenesis, preventing the unchecked formation of electrophysiologically silent excitatory synaptic co-clusters during early development [8].

Emerging evidence reveals that synapse-resident circRNAs can also serve as active translation templates, generating a novel landscape of functional micro-peptides directly within the synapto-dendritic arbor. High-throughput internal ribosomal entry site (IRES) reporter assays, combined with synaptosomal ribosome sequencing and mass spectrometry, have revealed that thousands of synapse-enriched circRNAs possess robust translational capacity that is dynamically modulated by behavioral experience [32]. The synapse-enriched *CircPcmtd1* is generated through back-splicing of a single exon from the *Pcmtd1* transcript and is highly expressed in response to learning. It contains an IRES with strong translation activity and generates the novel micro-peptide “P1”. Mechanistically, P1 is catalytically active and physically associates with plasticity-related proteins, including the core memory kinase CaMKIIa (Figure 2d). Functional electrophysiological profiling supports the idea that P1 modulates synaptic transmission, controlling the frequency of miniature excitatory postsynaptic currents in cortical neurons [32].

Besides the ones described, many circRNAs have been shown to contribute to synaptic regulation via different mechanisms (Table 1). For example, circTulp4 influences neurotransmitter release at excitatory synapses and aversive stimulus sensitivity [33].

These findings indicate that circRNAs are active components of synaptic regulatory networks. Through interactions with miRNAs, RBPs and RNA transport machinery, circRNAs contribute to the control of gene expression at synapses. Further studies will be essential to define how individual circRNAs integrate into signaling pathways and how their dysregulation may contribute to synaptic dysfunction.

## 5. CircRNA Transport Impairment in Neurodegeneration

Defects in RNA metabolism and axonal transport deficits are widely recognized as central pathological hallmarks of neurodegenerative diseases [44]. Because post-mitotic neurons are highly polarized cells with extensive structural architecture, they rely on long-distance transport mechanisms that ensure strict RNP and organelle trafficking to maintain spatial gene expression, local proteostasis, and synaptic plasticity. Axonal transport deficits are most likely early pathological events in such diseases and lead to the spatial mislocalization, somatic retention, or premature degradation of vital non-coding and coding transcripts, which gradually accelerate synaptic failure, loss of axonal integrity and eventual neuronal degeneration. The spatial distribution of circRNAs in neuronal sub-compartments, especially within dendrites and synaptosomes, and their ability to interact with RBP and RNAs (mRNAs and miRNAs) strongly suggests that deficits in neuronal transport may also impair localization of circRNAs and, therefore, the transport, local stability and translation of their targets.

One of the strongest mechanistic links connecting circRNA dysfunction to neurodegeneration lies in the extensive composition overlap between circRNA-associated and disease-causative RBPs. In fact, several RBPs involved in circRNA biogenesis and localization, including FMRP, FUS, and TDP-43, are also drivers of neurodegenerative pathologies.

In Amyotrophic Lateral Sclerosis (ALS) and frontotemporal lobar degeneration (FTLD), mutations in these RBPs trigger their dramatic nuclear-to-cytoplasmic translocation, leading to pathological phase separation, misfolding, and the formation of cytotoxic, insoluble inclusions [45]. This cytoplasmic aggregation causes a severe toxic gain-of-function by physically trapping key RNA species and halting their physiological movement along microtubule pathways [46].

**Figure 3:**
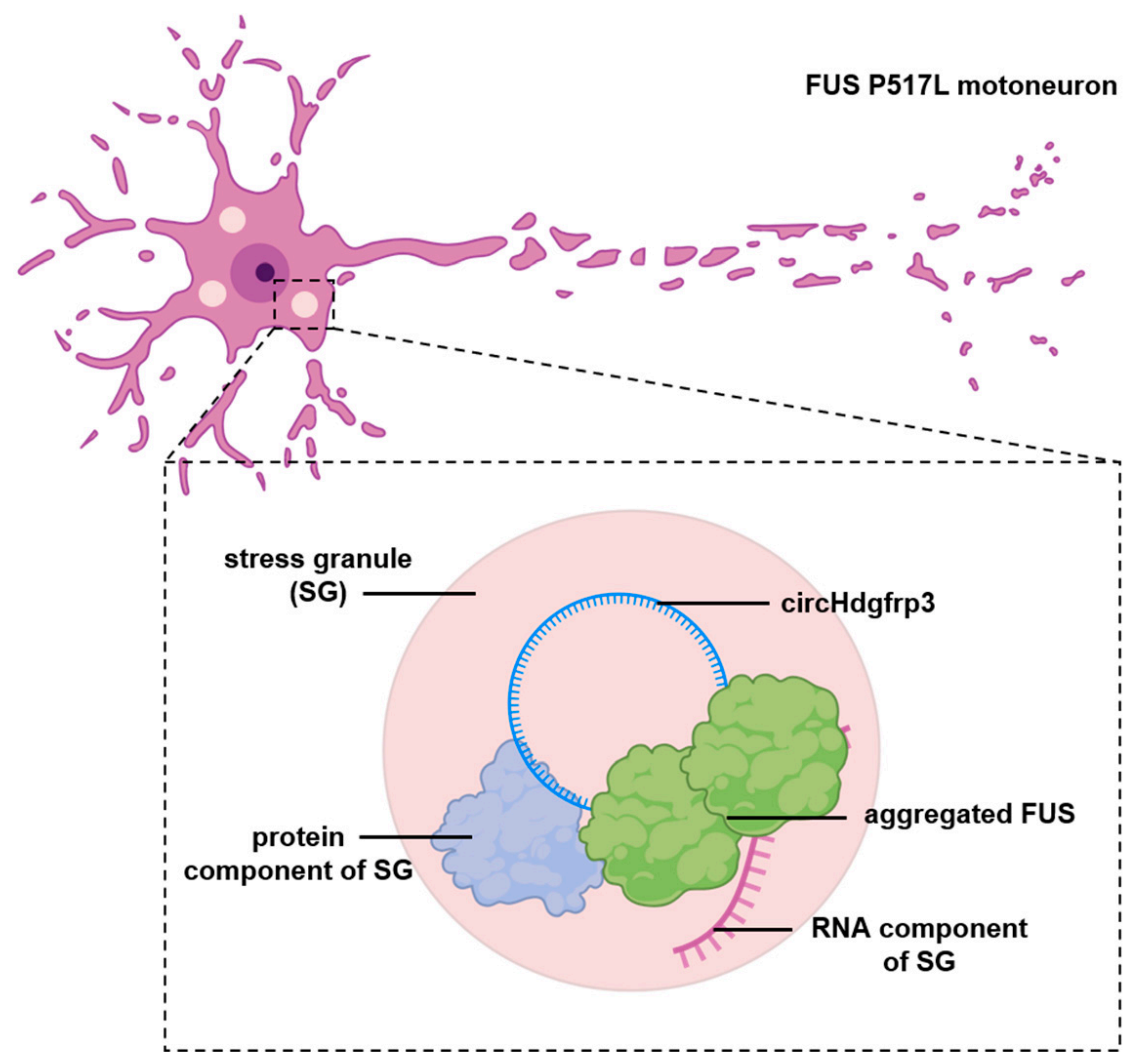
Schematic representation of *circHdgfrp3* sequestration in pathological FUS-containing RNP condensates under ALS conditions. In motoneurons carrying the ALS-associated *FUS P517L* mutation, aberrant cytoplasmic aggregation of FUS promotes the formation of persistent pathological granules. *CircHdgfrp3* is sequestered inside these granules, leading to alterations in its normal cellular distribution.

For instance, a clear, biochemically validated model of this transport failure involves *circ-Hdgfrp3* (derived from the *Hdgfrp3* gene), a brain-enriched circRNA highly expressed in murine motor neurons [47]. *Circ-Hdgfrp3* is upregulated during motor neuron differentiation and is enriched in neuronal processes. Notably, its biogenesis is controlled by the nuclear RBP FUS whose mutations are associated with familial forms of ALS. Mutant FUS undergoes cytoplasmic mislocalization and pathological aggregation, leading, among several other pathological alterations, to an impairment of RNA trafficking. Under basal physiological conditions, *circ-Hdgfrp3* is loaded into mobile RNP complexes and actively shuttles out into distal neurites where it may preserve local axonal homeostatic networks [48]. However, under conditions of oxidative stress, *circ-Hdgfrp3* is retained within the cell body in close proximity with stress granules. Wild-type cells smoothly liberate *circ-Hdgfrp3* back into the neuritic transport pool following stress removal. Critically, in motor neurons expressing ALS-associated mutant FUS, the persistence of immutable, mutant FUS aggregates even upon stress removal, irreversibly tethers and sequesters *circ-Hdgfrp3* inside cytoplasmic aggregates (Figure 3). This structural entrapment drastically hinders its spatial mobilization and prevents its delivery to distal neuronal processes, demonstrating how the physical immobilization of a circRNA may drive local functional deficits at the neuronal periphery.

Another example of circRNAs whose synaptic localization is altered in neurodegenerative conditions has been provided by Smukowski et al. [49]. In their study, they observed major differences in the synaptic composition of circRNAs in AD patients when compared to cognitively healthy controls. In particular, two isoforms of *circGSK3β* showed contrasting synaptic enrichment changes, with one being downregulated while the other was upregulated. The two circRNAs also seem to have an impact on the pathology; transfection of circRNA-expressing plasmids leads to downregulation of the phosphorylated form of Tau.

Although direct evidence for disease-associated circRNA mislocalization remains limited, some synaptic circRNAs, including *CDR1as* and *circHomer1*, are linked to regulatory networks known to be disrupted in neurodegeneration [13,15]. For example, chronic loss of *CDR1as* alters synaptic transmission stability and triggers neuropsychiatric deficits similar to early-stage cognitive decline [20], while the loss of spatial regulation over *circHomer1* variants disrupts spatial learning and memory control, consistent with its down-regulation across multiple neurological disorders [16].

Future work will be crucial to determine whether defects in circRNA transport pathways are primary driver of synaptic dysfunction or a secondary, downstream consequence of widespread global RNA metabolic failure.

## 6. Discussion

Over the past decade, circRNAs have shifted from being considered aberrant splicing by-products to being recognized as pivotal components of complex neural regulatory networks. The mammalian nervous system is particularly enriched in circRNAs, exhibiting a highly compartmentalized distribution across dendritic, axonal, and synaptic compartments. This selective localization, coupled with the remarkable stability of circRNAs and their dynamic responsiveness to neuronal activity, highlights their functional importance as active contributors to the compartmentalized regulation of gene expression, which underpins complex neuronal function.

In recent years, it has become evident that circRNAs are integrated into the molecular architecture of the synapse. Foundational studies of transcripts like *CDR1as*, *circRERE*, *circHomer1* and *circDlc1(2)* [8,16,20,21] have demonstrated that these circRNAs can modulate synaptic biology through different mechanisms, including the regulation of miRNA activity, the organization of RBP networks, and the modulation of excitatory synapse formation. This diversity in functions reflects the complexity of the synaptic environment, where multiple layers of post-transcriptional regulation are required to sustain local protein synthesis and neuronal plasticity.

There is also growing evidence that active circRNA trafficking may represent an essential mechanism of regulation, as passive diffusion alone cannot account for the marked enrichment of these transcripts in distal neurites. Recent findings, most notably the identification of *circHomer1* as a component of dendritic transport complexes [16], support a model in which circRNAs are incorporated into ribonucleoprotein particles and utilize cytoskeletal transport pathways. These observations place circRNAs within the framework of localized neuronal RNA biology, which has historically been centered almost exclusively on linear mRNAs [14,15]. Whether circRNAs harbor unique structure/localization signals or share a universal barcode system with linear transcripts remains an important area for future investigations.

Moreover, distinguishing active transport from local stabilization represents a significant challenge. Because circRNAs are highly resistant to exonucleolytic degradation, static localization measurements cannot discriminate among these possibilities. Addressing this question will require the development of approaches capable of simultaneously monitoring circRNA synthesis, transport, and turnover in living cells.

Compared with the extensive literature on mRNA transport and local translation, significant knowledge gaps remain regarding the mechanisms controlling circRNA trafficking. Only a handful of synaptically localized circRNAs have been functionally characterized, and transport mechanisms and physiological functions for the vast majority remain elusive. A major limitation is the lack of robust methodologies for tracking endogenous circRNAs in living neurons. Many observations on circRNA localization rely on endpoint analyses using fixed-cell methods such as single-molecule fluorescence in situ hybridization or on overexpression systems in which engineered circRNAs are tagged with fluorescent reporters [50]. While these methods have provided valuable insights into circRNA transport in neurons, they may not reflect the behavior of endogenous molecules. The development of improved live-cell imaging platforms and single-molecule detection tools will be critical to observe real-time circRNA transport, docking, and activity-dependent release within individual synaptic domains. An additional technical difficulty lies in distinguishing circular transcripts from their linear counterparts. Because circRNAs share nearly all of their sequence with the corresponding linear mRNAs, conventional imaging probes, RNA sequencing approaches, and spatial transcriptomic technologies frequently detect both forms unless specifically designed to target the back-splicing junction.

Finally, the involvement of circRNA transport pathways in neurodegeneration further underscores the importance of this field of study. Many proteins involved in circRNA trafficking and function, including the RBPs FUS, TDP-43 and FMRP, are central regulators of neuronal RNA metabolism and are associated with neurological disease [27,45,51]. Pathological mutations in these RBPs disrupt nucleocytoplasmic transport and alter liquid–liquid phase separation, causing them to form toxic cytoplasmic aggregates that halt intracellular trafficking highways. Moreover, several synaptic circRNAs have been linked to pathways disrupted in AD, PD, ALS and related disorders [20,48]. Although direct evidence for disease-associated circRNA mislocalization remains limited, the existing findings raise the possibility that perturbation of circRNA localization networks contributes to synaptic pathologies.

In conclusion, circRNAs are emerging as integral, dynamic components of neuronal RNA localization pathways and synaptic regulatory networks. Their enrichment in neuronal projections, incorporation into RNP transport systems, and the multifaceted functions they exert at the synapse point to a role in the spatial and temporal organization of gene expression within the nervous system. Future studies aimed at defining the molecular mechanisms and structural rules of circRNA trafficking, as well as tracking their mislocalization under pathological conditions, will undoubtedly reveal new layers of regulation in neuronal gene expression.

## Figures and Tables

**Table 1:** CircRNAs with a described function in synaptic regulation.

circRNA	Mechanism of Action	Reference
*CDR1as*	Sponges miR-7 to regulate synaptic protein expression and synaptic plasticity.	[30]
*circAnk3*	Sponges miR-7080-3p to regulate the structural plasticity of hippocampal synapses.	[34]
*circBptf*	Sponges miR-138-5p to regulate dendritic spine density and synapse-associated proteins expression.	[35]
*circDlc1(2)*	Regulates miR-130b-5p localization to regulate glutamatergic signaling.	[21]
*circFhit*	Recruits HNRNPK to enhance transcription of its parental gene, decreasing inhibitory synaptic transmission.	[36]
*circGRIA1*	Negatively regulates transcription of its parental gene, regulating synaptic plasticity and synaptogenesis.	[37]
*circHDAC9*	Sponges miR-138 to regulate synaptic function.	[38]
*circHomer1a*	Binds HuD to regulate alternative splicing and expression of synaptic plasticity genes.	[39]
*circ-Pank1*	Sponges miR-7a-5p, modulating the expression of α-synuclein.	[40]
*circPcmtd1*	Is translated into a small peptide that regulates synaptic activity	[32]
*circRERE*	Stabilizes miR-128-3p, negatively regulating excitatory synapses formation.	[8]
*circSatb2*	Promotes Robo3 transcription, regulating dendritic spine formation.	[41]
*circSYNDIG1*	Sponges miR-344-5p, regulating dendritic spine density.	[42]
*circTulp4*	Modulates the strength of excitatory neurotransmission.	[33]
*circVps41*	Sponges miR-26a-5p, regulating hippocampal synapses.	[43]

## Data Availability

No new data were created or analyzed in this study. Data sharing is not applicable to this article.

## Abbreviations

The following abbreviations are used in this manuscript:

AD	Alzheimer’s Disease
ALS	Amyotrophic Lateral Sclerosis
AMPA	α-Amino-3-hydroxy-5-methyl-4-isoxazolepropionic Acid
circRNA	Circular RNA
FMRP	Fragile X Messenger Ribonucleoprotein
FTLD	Frontotemporal Lobar Degeneration
FUS	Fused In Sarcoma
IRES	Internal Ribosome Entry Site
lncRNA	Long non-coding RNA
miRNA	Micro RNA
mRNA	Messenger RNA
PD	Parkinson’s Disease
RBP	RNA Binding Protein
RNP	Ribonucleoprotein
SMN	Survival of Motor Neuron
